# Live Birth in Woman With Premature Ovarian Insufficiency Receiving Ovarian Administration of Platelet-Rich Plasma (PRP) in Combination With Gonadotropin: A Case Report

**DOI:** 10.3389/fendo.2020.00050

**Published:** 2020-02-19

**Authors:** Chao-Chin Hsu, Leonard Hsu, Isabel Hsu, Yi-Jen Chiu, Sonam Dorjee

**Affiliations:** ^1^Taiwan United Birth-Promoting Experts (TUBE) Fertility Clinic, Tainan, Taiwan; ^2^Department of Obstetrics and Gynecology, National Taiwan University Hospital, Taipei, Taiwan; ^3^Nepean Hospital, Penrith, NSW, Australia

**Keywords:** premature ovarian insufficiency (POI), platelet-rich plasma (PRP), gonadotropin, ovarian administration, controlled ovarian stimulation (COS)

## Abstract

The conception rates among women with premature ovarian insufficiency (POI) remain extremely low. To achieve a successful pregnancy, most of these women have to receive donor oocytes through IVF treatment. Ovarian administration of platelet-rich plasma (PRP) has been recently applied to enhance the ovulatory function in women with poor ovarian response. However, no live birth has been reported for this application in patients with POI. In this study, we present a 37-year-old woman with POI who had secondary amenorrhea for 6 months. The clinical manifestations and evaluation of this women with a diminished ovarian function were an undetectable serum level of AMH (<0.02 ng/mL) and an elevated serum level of FSH (63.65 mIU/mL). A single dose of autologous PRP (extracted from 40 mL of peripheral blood) in combination with gonadotropin (150IU rFSH/75 IU rLH) was directly injected into the stroma of bilateral ovaries via vaginal sonographic guidance. Following the treatment, this patient received controlled ovarian stimulation and IVF during the successive months. Following embryo culture, three cleavage-stage embryos were transferred, leading to a successful pregnancy, which later resulted in the live birth of twins. This case report provides one example of alternative therapy that allows POI patients to use autologous oocytes in IVF treatment.

## Introduction

Premature ovarian failure or insufficiency (POF or POI) is characterized by the presence of primary or secondary amenorrhea for more than 4 months in women under 40 years of age with an elevated serum level of FSH (higher than 25 mIU/mL) (1). The average pregnancy rate in women with POI was <1:9200 (2), indicating that infertility is one of the most concerning problems of patients with POI. Notably, in the last 2 decades, most women with POI have achieved pregnancies by receiving IVF-treated donor eggs. Nevertheless, intermittent ovarian activities have been observed in patients with POI, and spontaneous resumption of ovarian function occurs in 25% of these patients (3, 4). More remarkably, studies have shown that, by using the ovarian biopsy, primordial and pre-antral follicles are frequently found in women diagnosed with POI (5). Because the prevalence of POI has risen from 1% to nearly 2% in the last few decades (6, 7), it is clinically urgent that a method that can stimulate and promote the growth of primordial and pre-antral follicles in patients with POI is developed.

Platelet-rich plasma (PRP), a preparation of autologous plasma, can supply a broad spectrum of growth factors at a high concentration, which is potent to the stimulation of tissue regeneration at the cellular level (8). Evidence from *in vitro* studies shows that PRP supports the viability and growth of human early pre-antral follicles (9). Direct injection of PRP into the stroma of poor responder ovaries leads to an increase in the number of follicles and eggs retrieved (10, 11). To date, only one case of biochemical pregnancy was reported in a woman with POI after treatment with PRP (12). We thus speculated that PRP-derived growth factors may not sufficiently stimulate the development of early follicles. Recent studies have indicated that follicular growth is regulated by subtle interactions between gonadotropins (FSH and LH) and local factors produced by the theca and granulosa cells (13). FSH receptors are expressed in granulosa cells at the primary follicle stage, and they are required for follicular development into the pre-antral stage (14). FSH activity also increased the primordial pool and enhanced the early follicle stock (15). LH triggers granulosa wall dissociation and cumulus expansion as well as oocyte nuclear maturation (13). We therefore designed this study using the direct injection of gonadotropins combined with PRP (“PRP/Gn”) into the ovarian stroma, aiming at restoring the ovulatory function of a woman with POI and evaluating the pregnancy outcome.

## Case Presentation

### Patient

A 37-year-old housewife woman, manifested with a low serum level of AMH (0.23 ng/mL) that was detected at age 33, had irregular menstrual periods with an interval of 3–6 months for more than 1 year. She had experienced secondary amenorrhea for 6 months. At the ages of 32 and 33 years, she received two cycles of IUI. However, both cycles were canceled because no available follicle was found in response to gonadotropin stimulation. Five months before the present PRP/Gn treatment, her serum level of AMH was barely detectable (<0.02 ng/mL), and the serum level of FSH was 43.50 mIU/mL. Prior to the treatment, this patient underwent a routine preoperative evaluation, including a Papanicolaou smear of uterine cervix. She was asked to refrain from hormonal therapy or any other adjunct treatments for at least 2 months. On the week of the PRP/Gn treatment, the serum levels of FSH and LH were 63.65 and 44.91 mIU/mL, respectively. No antral follicle of her ovaries was observed using ultrasonographic scanning, and no follicle growth was detected despite gonadotropin stimulation. No previous gynecologic or systemic malignancies were found, and thus no chemotherapy or radiotherapy had been carried out. The karyotype examination and fragile X carrier screening revealed normal. Her TSH was 1.65 uIU/mL and anti-TPO, anti-thyroglobulin antibodies, lupus anticoagulant, anticardiolipin IgG and IgM antibodies, and anti-β_2−_glycoprotein-I IgG and IgM antibodies were negative. No symptoms or signs of adrenal disease were noted.

### PRP/Gn Treatment

After thorough consultation of the benefits and risks of ovarian administration of PRP/Gn, the woman agreed to receive the treatment, and she gave written informed consent. This study was carried out according to the Declaration of Helsinki Good Clinical Practice and was approved by the local ethical committee (TSMH IRB/Protocol No: 18-115-B). Vaginal ultrasonographic scanning was performed one day before the treatment to ensure the status of the uterus and bilateral ovaries. PRP was prepared from 40 mL of peripheral blood by the buffy coat protocol (16). In brief, whole blood was divided into two aliquots and centrifuged at 3,000 rpm for 8 min at room temperature to form three layers: the bottom layer consisting of RBCs, the middle layer consisting of platelets and WBCs, and the top platelet poor plasma layer. The top platelet poor plasma and the middle layer were transferred to another two sterile tube and then centrifuged again at 4,000 rpm for 5 min. After removal of upper portion of the supernatant, each pellet was reconstituted with 2.5 ml of supernatant and was activated by adding 10% calcium gluconate before usage. The patient was lying in the dorsal lithotomy position, and a needle (Towako Transmyometrial Set 18 GA × 325 mm, Cook, Australia) was advanced into ovarian stroma under vaginal ultrasound guidance. A total of 5 ml of PRP combined with 1 ml of 150 IU of FSH/75 IU of LH (Pergoveris, MerckSerono, Aubonne, Switzerland) was slowly infused into ovarian tissue, 3 ml in each ovary, under intravenous sedation using 100 mg propofol (Fresenius Kabi Austria GmbH, Graz, Austria). No antibiotic was used, and the patient withstood the process without painful sensation. No visible side effect was noted in the whole procedure. As there was no previous information on the issue of intraovarian injection of Gn, we were more cautious of overdosage and used one vial of pergoveris that contained 150 IU of rFSH and 75 IU of rLH. The volume of PRP for intraovarian administration was done according to previous reports, and approximately 4 mL PRP per ovary was employed for the intraovarian injection (10, 17). After the PRP treatment, a 4-mm follicle was detected on day 4. It further grew into 10 mm on Day 8 with an elevated serum level of estradiol up to 136 pg/mL. The follicle did not grow larger but ovulated spontaneously, with the appearance of corpus luteum at right ovary and elevated serum progesterone of 46.78 ng/mL (Table 1). The menstrual cycle started at 25 and 48 days in the successive months after the treatment.

**Table 1:** The characteristics of hormonal profiles, follicles detected, oocytes retrieved, fertilized, and embryos transferred.

**Time of measurement**		**Hormone profile**				**Follicle size (mm)**		**Mature oocyte obtained**	**Fertilized eggs 2 PN**	Cleavage embryos/Grading (^*^)	**Embryo transfer**
		**FSH (IU/L)**	**LH (IU/L)**	**E2 (pg/mL)**	**P4 (ng/mL)**	**Lt**	**Rt**				
Month 0—treatment cycle	Before treatment	63.65	44.91	20	0.66			0			
	Day 4						4				
	Day 8			136			10				
	Day 11		97.15	104	0.79		10				
	Day 14		1.16	108	46.78		20 (^**^)				
Month 1 – menstrual cycle 1	Day 2	10.09	11.34	213	0.35	10	6	3	3	8-cell/4	ET
	Day 5					12, 6	10			5-cell/3	ET
	Day 9		15.87	552	0.6	14, 10	16			4-cell/3	
Month 2 – menstrual cycle 2	Day 2	17.84	3.43	76	0.18	7, 3	6, 6	3	3	8-cell/3	ET
	Day 5					8	9,7,5			6-cell/2	
	Day 9		4.44	830	0.41	10	15,15,11			4-cell/1	

* *Observed and graded (20) on Day 3, approximately 66 h after ICSI*.

** *Size of corpus luteum detected*.

### Controlled Ovarian Stimulation (COS)

In the two resumed menstrual cycles, the patient received COS with Gonal-F 300IU (Merck Serono) and Menopur 375IU (Ferring GmbH, Kiel, Germany) on day 2 and day 5 by intermittent vaginal administration (18, 19). In brief, the Gn was injected into superficial layer of vaginal mucosa at the middle to upper portions of the bilateral vaginal wall at positions corresponding approximately to 3 o'clock and 9 o'clock with the needle angled at 15–30° toward the vaginal mucosa and injected. A number of antral follicles were observed that continued to develop into mature follicles, and thus oocytes were retrieved on day 11 in both cycles. The characteristics of hormonal profiles, antral follicle counts, follicular growth, and mature oocytes retrieved are summarized in Table 1. Oocytes obtained from the first cycle were frozen and later thawed for fertilization. All six oocytes were fertilized with ICSI and cultured until day 3 stage embryos. Two 8-cell and one 5-cell stage good grading embryos, grade 4 representing regular spherical blastomeres with no extracellular fragmentation as the best embryo, were transferred back into the uterus (20). The serum level of β-hCG was 891.33 mIU/mL at day 13 after embryo transfer. A successful pregnancy with twins was confirmed by using ultrasonography. The preterm twins were delivered at gestational age of 30 weeks with a male newborn (body weight 1,300 gm) and a female newborn (body weight 1,258 gm). Both newborns thrived after birth. Currently, physical examination and mental development have been normal at postnatal regular follow-up checks.

## Discussion

Intraovarian PRP administration is a new therapeutic approach for patients with POI (21); however, the effect of PRP on regeneration of ovarian function does not seem to be optimistic, as only a handful of reports claimed a successful outcome (10, 11). In the present study, intraovarian PRP/Gn administration resulted in a successful pregnancy that gave rise to a live birth in a POI patient who used to be an extremely poor responder to exogenous gonadotropins. The results obtained from our study showed that this treatment may restore the ovulatory function since menstrual periods resumed and good antral follicles were observed in successive two cycles. The serum levels of FSH and LH dramatically decreased on day 2 during the following two cycles. The serum level of estradiol increased from 20 pg/mL to 552 and 830 pg/mL on hCG day of the two successive cycles (Table 1). All the physiological and biochemical changes suggested that the patient shifted from menopausal status toward the ovulatory status. These changes were probably due to the effects of intraovarian injection of FSH/LH in combination with PRP. Most remarkably, this is the first report of a live birth after intraovarian PRP administration in women with POI.

Intermittent ovarian activities have been observed in patients with POI, and spontaneous resumption of ovarian function occurs in 25% of these patients. Nevertheless, spontaneous follicular development would most likely result in single follicle maturation. In the present case, three and four antral follicles were detected, and all those developed into mature follicles following treatment at month 1 and 2, respectively. Therefore, the follicular development at the present case was more likely due to the therapeutic effect of PRP/Gn. As the mechanism of PRP/Gn action in folliculogenesis remains to be elucidated, we do not know why the estradiol level was elevated (213 pg/mL) on day 2 of following month in the early follicular phase.

Previous study proposed that PRP might enhance neoangiogenesis of menopausal status ovary and might promote the development of ovarian stem cells to mature follicles (10, 11). In the present study, intraovarian PRP/Gn administration appeared to restore the follicular development in antral, early antral, and preantral stages. Preovulatory maturation of antral follicles is very sensitive to gonadotropins. The follicles detected 4 days after the treatment, which spontaneously ovulated later, were likely resulted from a number of undetectable antral follicles before the treatment. Notably, serum levels of LH and FSH of the present subject were at a high level before the treatment, and the endogenous gonadotropins did not induce any maturation of follicles or ovulatory events in the past 6 months. Furthermore, three and four antral follicles were observed at 25 and 48 days post treatment in successive menstrual cycles, and these antral follicles were likely resulted from restored folliculogenesis of preantral or early antral follicles. Recent studies indicated that FSH promotes preantral follicular growth by acting in synergy with several intraovarian factors, such as C-type natriuretic peptide, kit ligand, leukemia inhibiting factor, and growth differentiation factor 9 (22). It is most likely that the direct ovarian injection of FSH interacts with local intraovarian factors that promote the basal follicular growth and development, while LH may further support follicle development by cumulus expansion and maturation of oocyte nucleus. Together with the subsequent COS, the combined effects gave rise to the development of mature oocytes. However, the roles played by FSH, LH, and PRP in restoration of ovarian folliculogenesis could not be clearly identified, as a combined treatment of PRP/Gn was used in the present case. Besides, since this woman conceived at 63 days post treatment and continued breastfeeding for more than 1 year after delivery, we were not able to examine whether PRP/Gn possibly stimulated the growth of ovarian stem cells or primordial, primary, or secondary follicles. For the same reason, the possible effect of PRP/Gn on AMH level was not followed in present study. Taken together, we speculate that ovarian administration of gonadotropin in combination with PRP may stimulate the growth and development of follicles from preantral to antral stages.

The current study raises a promising therapeutic strategy for direct administration of gonadotropins to its target organ, the ovary. To obtain multiple follicles for IVF treatment, daily injections of gonadotropins is especially stressful for infertile couples. Besides, routine peripheral administration of gonadotropins may only result in relatively poor bioavailability in the target organ (23). A recent pharmacokinetic study using a single subcutaneous abdominal injection showed that the absorption rates of recombinant FSH were low from the dosing site and the transit to central compartment which were 0.517 and 0.160, respectively (24). Furthermore, mixed literature is available regarding the aneuploidy rates in preimplantation genetic screening using array CGH or SNP in women receiving higher or lower doses of gonadotropins for the COS (25–27). With the aim of reducing the physical, emotional, and financial burden of patients, a more patient-friendly approach, including a lower dose of gonadotropins and a reduced number of injections, should be taken into account (19, 28, 29). In the present subject, direct injection of gonadotropins into the ovary induced restoration of the ovulatory function without any adverse effect. As far as we know, there is no previous work including animal studies employing the direct injection of Gn into ovary; thus, no data of safety issue can be provided. However, millions of oocyte pick-up (OPU) procedures are carried out each year; all OPU processes apply needle punctures in bilateral ovaries, but no specific safety issues have been raised besides the risk of infection or bleeding from the puncture. The only limitation of this administration mode is that an invasive approach has to be carried out under mild sedation. Nevertheless, the administration mode not only achieves the object of precision medicine but also costs less.

As PRP/Gn treatment was performed by direct injection into the ovary, effects of mechanical stimulation elicited by needle punctures cannot be completely excluded. Mechanical signaling pathways can be triggered by external or internal stress that may affect cellular behavior in the ovary. Recent studies indicated that ovarian fragmentation disrupts Hippo signaling, activates Akt pathway, and thus promotes activation of the primordial follicle (30, 31). However, it takes more than 300 days for the development of primordial follicles to mature. Thus, the resumption of ovulatory function in the present subject is less likely due to force-induced activation of primordial follicles. Whether the resumption of ovulatory function in the present subject is due to growth stimulating effect of PRP/Gn or alternative mechano-signaling pathways elicited by needle pinching requires future study.

As POI is a heterogeneous disease caused by different pathogenic mechanisms, it requires an individualized therapeutic strategy for each patient. Spontaneous ovulation and natural pregnancies in POI patients are very rare but still possible, especially for women aged below 30 (3, 32). The current report provides one example of alternative therapy for POI patients, particularly of more advanced age, who wish to use autologous oocytes in IVF cycles. Thus far, no adverse effect has been noted from studies employing intraovarian PRP administration; however, clinicians have to be very cautious of the possibility of detrimental outcomes, such as the development of ovarian neoplasm or any health threats. Furthermore, a large population prospective cohort study is mandatory to investigate how ovarian injection of PRP/Gn, FSH/LH, or FSH only can trigger the restoration of ovarian function and whether that can be a potential new mode of treatment.

## Ethics Statement

This study was carried out according to the Declaration of Helsinki and Good Clinical Practice. It was approved by the regulatory authorities and local ethical committee (TSMH IRB/Protocol No: 18-115-B). This subject signed the written informed consent to participate. Written informed consent was also obtained from the participant for the publication of this case report.

## Author Contributions

C-CH designed the project, performed the PRP treatment as well as the IVF process, and drafted the manuscript. LH designed the project and drafted the manuscript. IH contributed to the drafting of the manuscript. Y-JC contributed to the drafting of the manuscript. SD performed the preparation of the PRP.

### Conflict of Interest

The authors declare that the research was conducted in the absence of any commercial or financial relationships that could be construed as a potential conflict of interest.

## References

[1] European Society for Human R Embryology Guideline Group on POI Webber L Davies M Anderson R Bartlett J Braat D. ESHRE Guideline: management of women with premature ovarian insufficiency. Hum Reprod. (2016) 31:926–37. 10.1093/humrep/dew027 27008889

[2] Aiman J Smentek C. Premature ovarian failure. Obstet Gynecol. (1985) 66:9–14. 3925400

[3] Bidet M Bachelot A Bissauge E Golmard JL Gricourt S Dulon J. Resumption of ovarian function and pregnancies in 358 patients with premature ovarian failure. J Clin Endocrinol Metab. (2011) 96:3864–72. 10.1210/jc.2011-1038 21994953

[4] Bachelot A Nicolas C Bidet M Dulon J Leban M Golmard JL. Long-term outcome of ovarian function in women with intermittent premature ovarian insufficiency. Clin Endocrinol. (2017) 86:223–8. 10.1111/cen.13105 27177971

[5] Rebar RW Connolly HV. Clinical features of young women with hypergonadotropic amenorrhea. Fertil Steril. (1990) 53:804–10. 10.1016/S0015-0282(16)53513-4 2110072

[6] Coulam CB Adamson SC Annegers JF. Incidence of premature ovarian failure. Obstet Gynecol. (1986) 67:604–6. 3960433

[7] Lagergren K Hammar M Nedstrand E Bladh M Sydsjo G. The prevalence of primary ovarian insufficiency in Sweden; a national register study. BMC Womens Health. (2018) 18:175. 10.1186/s12905-018-0665-2 30359245 PMC6202813

[8] Wang TR Yan LY Yan J Lu CL Xia X Yin TL. Basic fibroblast growth factor promotes the development of human ovarian early follicles during growth in vitro. Hum Reprod. (2014) 29:568–76. 10.1093/humrep/det465 24408318

[9] Hosseini L Shirazi A Naderi MM Shams-Esfandabadi N Borjian Boroujeni S Sarvari A. Platelet-rich plasma promotes the development of isolated human primordial and primary follicles to the preantral stage. Reprod Biomed Online. (2017) 35:343–50. 10.1016/j.rbmo.2017.04.007 28756131

[10] Sfakianoudis K Simopoulou M Nitsos N Rapani A Pappas A Pantou A. Autologous platelet-rich plasma treatment enables pregnancy for a woman in premature menopause. J Clin Med. (2018) 8:1. 10.3390/jcm8010001 30577435 PMC6352170

[11] Sills ES Rickers NS Li X Palermo GD. First data on in vitro fertilization and blastocyst formation after intraovarian injection of calcium gluconate-activated autologous platelet rich plasma. Gynecol Endocrinol. (2018) 34:756–60. 10.1080/09513590.2018.1445219 29486615

[12] Sfakianoudis K Simopoulou M Nitsos N Rapani A Pantou A Vaxevanoglou T. A case series on platelet-rich plasma revolutionary management of poor responder patients. Gynecol Obstet Invest. (2019) 84:99–106. 10.1159/000491697 30134239

[13] Gougeon A. Human ovarian follicular development: from activation of resting follicles to preovulatory maturation. Ann Endocrinol. (2010) 71:132–43. 10.1016/j.ando.2010.02.021 20362973

[14] Oktay K Briggs D Gosden RG. Ontogeny of follicle-stimulating hormone receptor gene expression in isolated human ovarian follicles. J Clin Endocrinol Metab. (1997) 82:3748–51. 10.1210/jc.82.11.3748 9360535

[15] Allan CM Wang Y Jimenez M Marshan B Spaliviero J Illingworth P. Follicle-stimulating hormone increases primordial follicle reserve in mature female hypogonadal mice. J Endocrinol. (2006) 188:549–57. 10.1677/joe.1.06614 16522734

[16] Dhurat R Sukesh M. Principles and methods of preparation of platelet-rich plasma: a review and author's perspective. J Cutan Aesthet Surg. (2014) 7:189–97. 10.4103/0974-2077.150734 25722595 PMC4338460

[17] Chahla J Cinque ME Piuzzi NS Mannava S Geeslin AG Murray IR. A call for standardization in platelet-rich plasma preparation protocols and composition reporting: a systematic review of the clinical orthopaedic literature. J Bone Joint Surg Am. (2017) 99:1769–79. 10.2106/JBJS.16.01374 29040132

[18] Hsu CC Hsu CT Gu Q Wang ST. Intermittent vaginal injections of gonadotrophins for ovarian stimulation in IVF treatment. Reprod Biomed Online. (2008) 16:617–20. 10.1016/S1472-6483(10)60473-7 18492363

[19] Hsu CC Kuo HC Hsu CT Gu Q. The absorption and uptake of recombinant human follicle-stimulating hormone through vaginal subcutaneous injections–a pharmacokinetic study. Reprod Biol Endocrinol. (2009) 7:107. 10.1186/1477-7827-7-107 19807931 PMC2764710

[20] Bolton VN Hawes SM Taylor CT Parsons JH. Development of spare human preimplantation embryos *in vitro*: an analysis of the correlations among gross morphology, cleavage rates, and development to the blastocyst. J In Vitro Fert Embryo Transf. (1989) 6:30–5. 10.1007/BF01134578 2708875

[21] Pantos K Nitsos N Kokkali G Vaxevanoglou T Markomichali C Pantou A, editors. Ovarian rejuvenation and folliculogenesis reactivation in peri-menopausal women after autologous platelet-rich plasma treatment. In: Proceedings of the 32nd Annual Meeting of ESHRE, Helsinki, (2016).

[22] Hsueh AJ Kawamura K Cheng Y Fauser BC. Intraovarian control of early folliculogenesis. Endocr Rev. (2015) 36:1–24. 10.1210/er.2014-1020 25202833 PMC4309737

[23] Karlsson MO Wade JR Loumaye E Munafo A. The population pharmacokinetics of recombinant- and urinary-human follicle stimulating hormone in women. Br J Clin Pharmacol. (1998) 45:13–20. 10.1046/j.1365-2125.1998.00644.x 9489588 PMC1873998

[24] Rose TH Roshammar D Erichsen L Grundemar L Ottesen JT. Population pharmacokinetic modelling of FE 999049, a recombinant human follicle-stimulating hormone, in healthy women after single ascending doses. Drugs R D. (2016) 16:173–80. 10.1007/s40268-016-0129-9 27003895 PMC4875924

[25] Sachdeva K Upadhyay D Discutido R Varghese MM Albuz F Almekosh R. Low gonadotropin dosage reduces aneuploidy in human preimplantation embryos: first clinical study in a UAE population. Genet Test Mol Biomarkers. (2018) 22:630–4. 10.1089/gtmb.2018.0063 30199281

[26] Barash OO Hinckley MD Rosenbluth EM Ivani KA Weckstein LN High gonadotropin dosage does not affect euploidy and pregnancy rates in IVF PGS cycles with single embryo transfer. Hum Reprod. (2017) 32:2209–17. 10.1093/humrep/dex299 29040519

[27] Wu Q Li H Zhu Y Jiang W Lu J Wei D. Dosage of exogenous gonadotropins is not associated with blastocyst aneuploidy or live-birth rates in PGS cycles in Chinese women. Hum Reprod. (2018) 33:1875–82. 10.1093/humrep/dey270 30137360

[28] Alper MM Fauser BC. Ovarian stimulation protocols for IVF: is more better than less? Reprod Biomed Online. (2017) 34:345–53. 10.1016/j.rbmo.2017.01.010 28169189

[29] Hsu CC Kuo HC Hsu CT Gu Q. Abdominal mesotherapy injection extended the absorption of follicle-stimulating hormone. Fertil Steril. (2011) 95:2134–6, 2136.e1. 10.1016/j.fertnstert.2010.12.007 21208615

[30] Kawamura K Cheng Y Suzuki N Deguchi M Sato Y Takae S. Hippo signaling disruption and Akt stimulation of ovarian follicles for infertility treatment. Proc Natl Acad Sci USA. (2013) 110:17474–9. 10.1073/pnas.1312830110 24082083 PMC3808580

[31] Li J Kawamura K Cheng Y Liu S Klein C Liu S. Activation of dormant ovarian follicles to generate mature eggs. Proc Natl Acad Sci USA. (2010) 107:10280–4. 10.1073/pnas.1001198107 20479243 PMC2890455

[32] Fraison E Crawford G Casper G Harris V Ledger W. Pregnancy following diagnosis of premature ovarian insufficiency: a systematic review. Reprod Biomed Online. (2019) 39:467–76. 10.1016/j.rbmo.2019.04.019 31279714

